# Beyond Healing: Embracing the Triple Bottom Line Approach in Post-pandemic Healthcare

**DOI:** 10.7759/cureus.54019

**Published:** 2024-02-11

**Authors:** Mairy Alim, Saanie Sulley

**Affiliations:** 1 Faculty of Economics and Management Sciences, University of Bamenda, Bamenda, CMR; 2 Health and Biomedical Informatics, Independent Researcher, Washington, USA

**Keywords:** pandemic preparedness insights, healthcare policy and management, equity in healthcare, green healthcare, healthcare resilience, sustainable healthcare metrics, tbl in post-pandemic healthcare, healthcare sustainability

## Abstract

A triple bottom line (TBL) encompasses economic, social, and environmental dimensions, which provides a strategy for transforming contemporary healthcare systems. This study contextualized current TBL developments in healthcare under the spotlight of COVID-19 pandemic-related challenges and opportunities. The paper has five sections, including an introductory section that outlines the TBL concept and its relevance to healthcare. Following this, we provide an overview of the three pillars of the TBL, including its economic, social, and environmental dimensions as they relate to healthcare. This section also includes several case studies to illustrate TBL-based practices in diverse healthcare settings, focusing on how these were implemented and the outcomes and barriers to adopting such practices. In addition to showcasing current TBL practices, we review three significant challenges to TBL and highlight potential areas for future research, such as innovative practices, educational reform, and the need for the development of robust TBL metrics. The overarching conclusion is that the TBL provides a profound approach to conceptualizing contemporary healthcare to meet the urgent requirements for a more resilient, equitable, and environmentally responsible healthcare system.

## Introduction and background

The triple bottom line (TBL), first coined by John Elkington in 1994, has been the 'economic spine' ingrained in sustainable development since the 1980s [1]. Initially emerging in the business context, the TBL, which provides a three-dimensional lens of social, environmental, and economic sustainability performance, has been increasingly adopted in different areas of life with different applications and challenging issues. Healthcare in the context of modern and highly complex health systems.

The practical application of TBL in healthcare recognizes a more holistic set of responsibilities than being profitable to stakeholders. Often promoted by governments, the integration of TBL in healthcare is based on the importance of moving toward a more sustainable model of healthcare delivery, one that increases the level at which there is interconnection between these three pillars of the triple bottom line. All recommendations emphasize a so-called triple-win model, in which global and national economic and social development goals can be realized only when they work in synergy with environmental sustainability goals.

Economic sustainability means using resources efficiently and responsibly, financing care and paying for it somewhat, and maintaining financial resilience so that health systems remain a source of ongoing value [2]. Social sustainability means that care is equitable and fair, that care delivery improves patient outcomes, respects the workforce’s time and satisfaction, and contributes to community well-being [3]. Environmental sustainability means reducing the overall carbon footprint and ecological impact of health systems and their operations, including, but not limited to, managing waste products, using energy, procuring supplies and pharmaceuticals, and food [4].

While implementing TBL in healthcare is not without significant hurdles, pursuing TBL objectives represents a potentially transformative agenda for healthcare organizations. Such organizations can pivot, or perhaps reimagine, their societal role: through TBL, the health sector embraces a collective orientation toward a platform of health and well-being where innovative public policies better respond to upstream social, economic, and environmental determinants.

Healthcare context and relevance of the triple bottom line

The TBL is crucial to modern healthcare, and it has become more relevant because health services operate at the intersection of social welfare, economic stability, and environmental impact. The modern health service industry focuses on service delivery, primarily patient care. Therefore, extending the reach of the modern healthcare system is imperative and should include TBL sustainability. The value of TBL for healthcare is, therefore, several-fold. First, economically, healthcare is a massive contributor to the gross domestic product (GDP) in most countries [5]. In addition to the ethical responsibilities healthcare systems incur in fulfilling their mission to care for people, they also have an 'economic responsibility' to use resources wisely to sustain their financial viability over the long term while continuing to improve quality and outcomes.

From a societal perspective, healthcare systems are central to well-being in society, as was clearly shown by the COVID-19 pandemic, which was as much a healthcare issue as a public health issue, and healthcare services were necessary not only for those who developed severe symptoms but also as a critical pillar of societal resilience. It also highlighted the vast inequalities in access to health services. Therefore, TBL calls for a systems approach to healthcare that encompasses both health outcomes and social determinants of health. Environmentally, it is a highly resource-intensive sector that generates a large amount of carbon emissions and waste [6]. Another area where TBL could be applied is to enable the healthcare organization or environment and the patients of the service to minimize the ecological footprint.

TBL could also support business strategy and technological innovation and development, as fostering more sustainable business models is crucial. Indeed, TBL has helped businesses return profits by adopting sustainable business models, where consumer positivity and cost savings are the main factors [7]. Therefore, in the healthcare context, TBL becomes more critical to address in response to present and future stakeholder demands because they all represent vital parts of the healthcare ecosystem and ecosystem services. Healthcare stakeholders demand more responsible business practices and, ultimately, more sustainable healthcare services. The same is true in the context of broader societal expectations of sustainability and corporate social responsibility.

## Review

Economic sustainability in healthcare

It is critically important to ensure economic sustainability, which is the second cornerstone of the TBL. It is impossible to implement TBL when the third cornerstone does not exist, when the focus of value creation is not predominant, and when the value we generate is no longer about the informal value we give generously to people and things we care about in the end. Health is a natural value of life if we consider it from the perspective of the informal value that we freely give to people and things that are important to us. These are the foundations of this aspect of TBL.

A second key pillar as far as financial sustainability is related to what we refer to as value-based care [8], which describes how moving from a volume-based to a value-based approach to healthcare delivery might improve how we pay for and deliver care. In a volume-based setting, providers receive payments for services rather than payments for patients' health outcomes. For example, the amount paid for medical care to a diabetic patient may include payment for long-term education to avoid future complications, not just for a hospital visit to the emergency department. Even if clinicians attempt to reduce visits, unnecessary ancillary services, and low-cost generic drugs, such efforts may not translate into economic gains because payments for the income associated with disease and medical activities are diversified according to clinical value.

Therefore, a volume-based incentive goes against promoting good health because providers are given incentives to provide care to maximize income associated with disease and to incentivize diversifying activities according to the value of clinical care. In contrast, in a value-based care setting, paying for outcomes would encourage the adoption of innovations that improve quality and efficiency, reduce tests, and eliminate waste and redundant activity, thereby aligning economic incentives with utility for the patient. A second key feature of the financial sustainability of healthcare is ensuring the efficient use of inputs to maximize outputs. These include inputs in health and non-health.

All these inputs must be provided in the correct quantity and quality while not generating unnecessary improvidence. Examples of these approaches to securing the financial sustainability of healthcare include lean management in Japan for reducing waste and economic sustainability. TBL's economic pillar speaks to more than just preserving financial viability. At its core is a reconceptualization of 'economic success' as having a more significant impact relative to patient health and the broader 'triple bottom line' of the healthcare system.

Social sustainability in healthcare

Social sustainability, as the second of the three dimensions of the TBL in health, includes everything from the quality of patient care to the health of care workers and their families to the effect of healthcare systems on the population they serve. This dimension focuses on the social contract between healthcare systems and communities and prioritizes population approaches to health. A crucial aspect of social sustainability in healthcare, equitable access to healthcare services, implies addressing disparities in use and quality of care that are far too often deeply rooted in fundamental social determinants of health. Targeting these 'social iniquities' of health rather than adopting medical model approaches is an ethical and necessary endeavor since social and economic conditions are significant determinants of collective health and well-being.

Quality of patient care might include ensuring patient safety, improving patient's healthcare experience, and delivering good health outcomes. Programs that promote patient-centered care and integrative programs that combine holistic health services are a step toward a more socially sustainable health system. Workforce well-being is also a critical element of social sustainability in healthcare. Healthcare professionals are often among those most impacted by poor health, and low morale can lead to lower-quality care. Taking steps such as reducing physician burnout, ensuring that healthcare providers receive compensation, and creating safe workplaces are all important in sustaining a healthy and productive healthcare workforce. Longitudinal research commissioned by the Mayo Clinic [9] found that the scale and scope of physician burnout are alarming.

Moreover, healthcare organizations should participate in community engagement and public health programs to assume social responsibility. By joining more community health programs, health education and community organization programs can become examples of social sustainability. Second, social sustainability is about creating an environment in which the health needs of people and communities are fair and integrated, resulting in safe, functioning, and sustainable societies.

Environmental sustainability in healthcare

It specifically refers to the environmental impact of healthcare provision, i.e., the ecological footprint of healthcare provision. This aspect of TBL focuses on minimizing the ecological footprint of healthcare provision. It relies on the fact that there is a direct link between ecological health and public health outcomes. Healthcare activity contributes to environmental pollution and the use of resources. For example, healthcare accounts for approximately 5%-10% of national carbon emissions in many countries [10], so the environmental side of TBL for healthcare might concern efforts to reduce carbon footprints, responsibly use waste, and employ energy-efficiency measures.

Waste management is crucial because of healthcare facilities' vast medical and non-medical waste. Moreover, environmentally conscious and sustainable waste management principles are vital in ensuring the utilization of waste through recycling, disposal of hazardous waste, and reduction of single-use medical supplies [11].

Another significant issue that requires attention is the energy used in healthcare facilities. Hospitals are known as large-emission energy-use industries. Energy-saving devices or technologies, such as LED lighting and heating and cooling systems, have been widely explored because they improve energy efficiency and reduce pollution and its adverse effects [4]. Furthermore, sustainable procurement management serves environmental responsibility by selecting suppliers and goods that comply with environmental standards, such as minimal packing and non-toxic materials in the supply chain [6]. Integrating environmental sustainability into healthcare operations reduces ecological injuries and enhances health outcomes. There is a heightened awareness that environmental health is the basis for public health and that environmental stewardship is part of healthcare delivery.

Impact of COVID-19 on the need for TBL in healthcare

The COVID-19 pandemic serves as an inflection point that has reiterated the need to embrace the TBL in healthcare to achieve comprehensive sustainability. The COVID-19 pandemic is unique in many ways, but what is distinct here is the fact that it has connected the dots that healthcare, the economy, and ecology have a shared fate and that we will erode economic stability, social equity, and environmental health by driving ecological loss and inequality.

The unexpected surge in healthcare demand and interruption of standard services brought about by the pandemic has put the economic sustainability of the health sector at risk. Recent experience has demonstrated that the financial sustainability of health services is crucial to the ability of healthcare systems to continue providing essential services in extraordinary circumstances. In the aftermath of the pandemic, healthcare policymakers should consider adopting new economic models to make health systems more resilient to shocks.

COVID-19 has caused or worsened severe disparities in healthcare use and outcomes, such that the right to health of vulnerable populations is at risk of being violated. It has been made glaringly obvious that, with an urgent prioritization of the social aspects of health as well as access to health systems, there will always be equity in healthcare use and outcomes. The pandemic brought the issue of social sustainability right to the fore of the health policy agenda, helping to re-center attention on health as a universal basic social entitlement, implying the need to make health care inclusive and equitable [12].

TBL and sustainability, more generally, are linked to the response to the pandemic. Notably, concerns linked to environmental sustainability issues around TBL have also been raised alongside the pandemic, mainly due to issues around the production and management of medical waste and disposal solutions, as well as policies for resource provision within the domain of the response to the pandemic. Indeed, the quantity and complexity of medical waste, linked to the increased demand for resources due to the pandemic, put the issue of environmental sustainability at the focus of healthcare, with new criminality and new modalities of sustainable disposal [13].

We have learned many lessons on anchoring TBL more firmly into the health system from our experiences with COVID-19. To be better prepared for future health emergencies, such health systems should be economically shockproof, equitable for all, environmentally sustainable, and adaptable to new global realities.

In the past year or two, pandemic experiences have underscored the systemic fragility of global healthcare. They demonstrate the necessity of a TBL approach that addresses current healthcare needs and knowledge about the long-term determinants of society and the environment.

Case studies and best practices

Real-world case studies and best practices are indispensable in gaining a broader understanding of potential approaches. They make the TBL approach in healthcare natural and provide valuable illustrations of where and how it works. They depict the real challenges and success stories of healthcare organizations seeking sustainability.

Kaiser Permanente is one of the largest not-for-profit healthcare organizations in the United States. It has led the way in efforts to go green, and this commitment and achievement is the hallmark of their TBL approach. Kaiser Permanente became carbon neutral in 2020, an early milestone achieved as part of the company's comprehensive stewardship plan for carbon, water, and sustainable food procurement [14].

The Cleveland Clinic's experience has been based on several programs addressing sustainability's social and environmental facets. From community health improvement programs to hundreds of energy conservation measures, the organization's overall efforts have reduced its carbon footprint, sponsored numerous community service events and hydroponic gardening, and saved millions of dollars [15].

Sweden is renowned for waste and energy efficiency. Waste is efficiently recycled in hospitals in Sweden, and many of these sites use renewable energy-a prime example of environmental sustainability within healthcare systems [16].

Value-based care initiatives have advanced economic sustainability for Partners HealthCare (now Mass General Brigham) by sharing cost savings to improve patient outcomes. Their workforce development programs in the above area have invested in healthcare professionals staying skilled and satisfied in their jobs to be socially sustainable [17].

Community Health Initiatives at Johns Hopkins Hospital in Baltimore, Maryland, is a notable example of how the TBL extends its reach towards social sustainability. Johns Hopkins Hospital has invested in community health, including health education, free clinics, and other programs that address the social determinants of health in urban communities [18]. Improving the health outcomes of society is an essential component of the TBL in healthcare.

Gundersen Health System, based in La Crosse, Wisconsin, has attained Energy Independence. Through a combination of energy efficiency investments and energy conservation actions, the system has saved money on energy and has designed facilities that significantly reduce its environmental impact. They have achieved a balanced approach between the environment and the economy [19].

Narayana Health is a network of hospitals across India that exemplifies economic sustainability when high-quality, affordable care is delivered using good operations efficiency and simplicity with a focus on affordability. Narayana Health demonstrates the financial viability and sustainability of an ethical and socially responsible business model that attempts to reach the unserved population, focusing on high-quality, affordable health services [20].

Many observers have acknowledged that the healthcare system in Singapore is efficient. The preventive managed care model is the key; the government has invested in health technology to enhance sustainable practice. In addition, quality and efficiency are mutually exclusive in the national healthcare system, which benefits economic and social sustainability. People can enjoy high-quality medical services at a low cost [21]. The overall healthcare system in Singapore is high in efficiency and sustainability. Furthermore, the government has been investing in health technology, such as machine learning and aid algorithms, successfully innovating tertiary healthcare at a low cost and high quality. It demonstrates its efficiency and sustainability to the national healthcare system; insured people can enjoy high-quality medical services.

Policy and regulatory frameworks

Policy and regulatory frameworks play a central role in implementing the triple bottom line (TBL) in healthcare settings that can facilitate or impede the pursuit of TBL. Successful pursuit of TBL in healthcare settings requires a strong understanding of the policies and regulations related to healthcare.

Government policies and mandates can accelerate and drive this agenda. Policies or legislation promoting acts of 'greening' such as subsidies for using renewable energy in hospitals and creating standards or regulations to cut healthcare waste can promote sustainability. Moreover, the Affordable Care Act in the U.S., also known as Obamacare, brought in a slew of measures designed to improve the efficiency and quality of the healthcare sector, hence indirectly advancing TBL's economic and social aspects [22].

International Agreements and Standards International agreements, such as the Paris Agreement, for example, can affect healthcare sustainability via the indirect effect of national policy. Many of these agreements target and encourage carbon reduction, which inevitably includes the healthcare sector. International standards on environmental management, such as the International Organization for Standardization (ISO) 14001, can also influence healthcare systems and facilities to reduce their environmental impact [23].

Local and regional healthcare regulations could evolve to address the TBL, addressing items such as local waste management, energy use, and community health programs, which could help healthcare organizations pursue their activities.

Even with supportive policies, implementing new care models in healthcare organizations can be problematic and burdensome. Problems with bureaucracy, lack of clarity in regulations, or insufficient financial resources or staffing levels can hinder achieving sustainability goals.

Standards set by accreditation and certification bodies can encourage TBL. For example, the green building certification of Leadership in Energy and Environmental Design (LEED) is a rating system that certifies healthcare facilities based on sustainability in their design and construction.

Even the dynamic interaction between policy, regulation, and healthcare sustainability underlines the need for harmonious implementation of TBL in healthcare. For the TBL to truly work in improving health outcomes, creating healthy environments, and achieving a sustainable future, health policies and regulations described by the government must be implemented by healthcare organizations and supported by the active participation of critical stakeholders.

Measuring and reporting TBL in healthcare

The measurement and reporting of TBL are essential for accountability and transparency regarding the effectiveness of sustainability efforts. Solid metrics and reporting approaches are fundamental to helping healthcare organizations monitor their progress in the TBL for themselves and their stakeholders.

Measuring TBL in healthcare requires precise and reliable metrics. Creating metrics to measure economic sustainability might focus on cost-effectiveness, growth and revenue, and the rates of scarce resource utilization. For example, the use per square foot can indicate the efficiency of a hospital's operation. To measure social sustainability in healthcare, metrics include patient satisfaction scores, discrepancies in health outcomes across population segments, and employee engagement levels. Environmental metrics might focus primarily on metrics that measure carbon footprint, reduction of waste and recycling, and energy efficiency [24].

Balanced scorecards could be a powerful measurement method for TBL through periodic reporting that allows organizations to simultaneously chart performance in economic, social, and environmental domains. The measurement would assist in aligning sustainability goals with organizational strategy and balancing competing elements of TBL [25].

Regular and transparent reporting of TBL outcomes is required as a reporting mechanism for accountability. Annual sustainability reports, usually aligned with a reporting standard such as the Global Reporting Initiative (GRI), cover the organization's sustainability performance, including achievements and where shortfalls are observed [26].

Providing standardized metrics to measure TBL in healthcare is challenging due to the diversity of variables. Measuring and reporting performance is often complex and inconsistent. This highlights the need for more standardization in reporting and transparency; there was a compromise in benchmarking.

Technology has a significant role in TBL reporting, such as in health informatics and data analytics. TBL is progressing in tandem with technology, allowing for better data collection, analysis, and dissemination, resulting in its ability to serve as a better metric. Ultimately, measurement and reporting are fundamental to the success of TBL in healthcare because they showcase accountability, advance reporting on performance across the three dimensions of people, planet, and profit, and identify where additional efforts are needed to improve any of these dimensions.

Challenges and barriers

The introduction of TBL in healthcare faces many challenges and barriers. There are different types of constraints: organizational, economic, social, and environmental. These barriers are essential to consider to make TBL in healthcare palatable conditions.

The first challenge is more about financial means and healthcare departments, especially in contexts where resource limitations are more apparent. Organizations might need more time to dedicate resources toward sustainability in competition with clinical needs [27].

Cultural and behavioral change: TBL often entails substantial cultural and behavioral shifts within health organizations; pushback from staff and management can make shifting to more environmentally sustainable working methods challenging. This is particularly true in social sustainability, which involves shifts in organizational culture and mindset [28].

There may be ignorance of TBL and, thus, a lack of willingness to act or a misunderstanding of the reasonability of TBL on behalf of healthcare professionals. The lack of awareness might be due to the intricacy of the formulation of TBL concepts, which demand a certain level of expertise by healthcare professionals that might only exist in some healthcare organizations [24].

Inadequate and restrictive regulations and policies can hamper inherent attributes. TBL businesses also face additional barriers that prevent them from harnessing their human, intellectual, and social capital. One such obstacle lies in the regulatory and policy contexts that hinder the adoption of TBL practices. This obstacle is particularly relevant in environmental sustainability because regulatory frameworks are vital in shaping and enforcing sustainability [29].

Developing metrics for TBL outcomes and implementing standardized reporting of activities and outcomes across various socially relevant dimensions can pose considerable challenges. In addition, social and environmental outcomes, sometimes less tangible than economic results, are more challenging to measure and report than economic outcomes [25].

Balancing TBL's economic, social, and environmental aspects may be challenging. Profitability, ethical patient care, and ecological thoughtfulness often conflict in healthcare settings [30] and can limit their balance.

Combating these requires planning, education, training, stakeholder engagement, and sometimes lobbying policy. Health institutions must acknowledge where they face roadblocks and devise ways to overcome them.

Future directions and research needs

Future direction and research priorities are also paramount as healthcare increasingly embraces the TBL paradigm. The TBL concept continues to patiently and consistently evolve and will do so for the foreseeable future, as further innovations, research, and policy work make many new opportunities and challenges visible and possible.

Emerging strategies with feasible actions to advance the sustainability of healthcare will be necessary to structure future research and innovation to foster novel technical and social interventions that are environmentally sound, improve social outcomes, and enable a health-promoting, financially, and economically sustainable healthcare system in both high- and low-income settings. This includes introducing novel digital health, eHealth, and mHealth solutions, devices, infrastructure, green hospitals, and research platforms.

As healthcare continues to trend towards an era of sustainability, integrating TBL concepts into medical and health professional education is needed. The research seeks to develop and assess health university curricula with TBL concepts to best prepare healthcare providers with holistic approaches to the environment.

A compelling domain for future research could be designing the standards or metrics to measure TBL outcomes. This research can help develop a commonly accepted set of indicators for comparing and benchmarking performance across healthcare settings and systems.

Research on policies promoting TBL in health research could continue. The literature on environmental policies meant to reduce mining emissions appears in economics and environmental science; therefore, comparable research into whether those policies are applicable in health and to promote TBL in health care would continue and push for additional policies that are more enabling of TBL practices.

Besides investigating the individual dimensions of TBL, future research should also examine the interdependencies between the three dimensions of economic, social, and environmental criteria. Developing strategies to balance the mutual relationship between these dimensions to address complex healthcare environments is essential.

Longitudinal studies of TBL initiatives in healthcare are needed to determine their long-term impacts. Such studies can determine if TBL can be sustained and recommendations can be implemented. They can also inform alterations and modifications of TBL practices over time. The future of TBL in healthcare is complex and exciting. Addressing these research gaps is necessary to help healthcare systems reach the triple bottom line to advance health outcomes for people, society, and the planet.

## Conclusions

As we examine the triple bottom line in healthcare, we find that it takes on an entirely new meaning, a means of healthcare delivery that goes beyond the traditional delivery model by focusing on economics, people, and the environment. The TBL is a model for healthcare organizations to become economically viable by delivering safe and high-quality service, contributing to societal health and well-being, and being an agent of environmental health and ecological protection.

This review argues that TBL principles are integral to healthcare systems. Broadly, economic sustainability means being able to deliver healthcare services at the right volume, with the correct cost, for the suitable duration, for the right members of our community, at the right moment, and in a manner that produces stable financial results and appropriate utilization of healthcare resources. It is often the first pillar that is mentioned and discussed. Social sustainability refers to the healthcare system delivering healthcare in a way that offers equitable access to care for members of our community in healthy environments where there is ethical care toward those who receive it. This means delivering healthcare in a way that will have a positive impact on society, which counts. Environmental sustainability pertains to reducing the ecological footprint of the operation of our healthcare systems, which is an essential step to public health and the planet to protect the health of our future generations.

As this review demonstrates, while challenging, it can be implemented in healthcare, and the evidence of benefits will be helpful to other healthcare organizations as they try to infuse their operations with values. Public Health England and ESCo, an NHS Confederation initiative, have been supplied with many of these studies as well as numerous best practices. They are trying to use what they learn to reform healthcare for drama and reality, a blueprint for clinical excellence.

However, we can move further with continued innovation, further research, and policy development to operationalize the TBL framework to healthcare, finding new forms of practice that work, new metrics that are relevant and useful, and building the reflex of looking to fundamental economic, social, and environmental imperatives in everything we do within the healthcare system. The triple bottom line provides a framework for radically reorienting the values in how we organize health services to be more aligned with broader social and environmental aims. Moreover, given the stakes, it would be unethical not to start doing so today.
